# Effects of Climatic Change on Potential Distribution of *Spogostylum ocyale* (Diptera: Bombyliidae) in the Middle East Using Maxent Modelling

**DOI:** 10.3390/insects14020120

**Published:** 2023-01-24

**Authors:** Mustafa M. Soliman, Areej A. Al-Khalaf, Magdi S. A. El-Hawagry

**Affiliations:** 1 Department of Entomology, Faculty of Science, Cairo University, Giza 12613, Egypt; 2 Biology Department, College of Science, Princess Nourah bint Abdulrahman University, Riyadh 11671, Saudi Arabia

**Keywords:** species distribution model, parasitoid bee fly, conservation, maxent, climate change

## Abstract

**Simple Summary:**

*Spogostylum ocyale* (Wiedemann 1828) is a robust species of bee fly (family Bombyliidae), known to be a larval ectoparasitoid as well as an important flower pollinator. This species has disappeared from many of its historic habitats due to substantial changes in floral and faunal compositions in recent years. The current and future distributions of the parasitoid in the Middle East region was predicted using the Maximum entropy model (Maxent). The model performance was satisfactory and revealed a good potential distribution for *S. ocyale* featured by the selected factors. The results show that the distribution of *S. ocyale* is mainly affected by the temperature. Coastal regions, with warm summers and cold winters, were high to medium in suitability. Future scenarios predict a progressive decline in the extent of suitable habitats with global warming. These findings lead to robust conservation management measures in current or future conservation planning.

**Abstract:**

*Spogostylum ocyale* (Wiedemann 1828) is a large robust species of bee fly (family Bombyliidae), known to be a larval ectoparasitoid as well as an important flower pollinator as an adult. This species has become extremely rare or has disappeared from many of its historic habitats due to substantial changes in floral and faunal compositions in recent years. Climate change and urbanisation, together with other anthropogenic activities, may be to blame for these changes. Distribution modelling based on environmental variables together with known occurrences is a powerful tool in analytical biology, with applications in ecology, evolution, conservation management, epidemiology and other fields. Based on climatological and topographic data, the current and future distributions of the parasitoid in the Middle East region was predicted using the maximum entropy model (Maxent). The model performance was satisfactory (AUC mean = 0.834; TSS mean = 0.606) and revealed a good potential distribution for *S. ocyale* featured by the selected factors. A set of seven predictors was chosen from 19 bioclimatic variables and one topographic variable. The results show that the distribution of *S. ocyale* is mainly affected by the maximum temperature of the warmest period (Bio5) and temperature annual range (Bio7). According to the habitat suitability map, coastal regions with warm summers and cold winters had high to medium suitability. However, future scenarios predict a progressive decline in the extent of suitable habitats with global climate warming. These findings lead to robust conservation management measures in current or future conservation planning.

## 1. Introduction

*Spogostylum ocyale* (Wiedemann 1828) is a large robust species of bee fly (family Bombyliidae), about 15 mm in length or slightly more [1]. Like all *Spogostylum* spp., adult *S. ocyale* are important flower pollinators [2], while as larvae they are ectoparasitoids on the immature stages of solitary bees and wasps, egg pods of Acrididae and pyralid cocoons [3,4]. The distribution of *S. ocyale* is primarily restricted to warm arid and semiarid habitats [5,6], where it is a prominent member of the flower-visiting insect fauna [7]. It has been recorded and seems to be well represented throughout most Middle Eastern countries, as well as a few neighbouring countries, in both the Palaearctic (Egypt, Iran, Israel, Jordan, Libya, Palestine (West Bank) and Saudi Arabia) and Afrotropics (Oman, Somalia, southwestern Saudi Arabia, Sudan, United Arab Emirates and Yemen) [8].

The distribution of a living organism is a necessary component of the study of its occupied ecological niche in the ecosystem [9]. The niche of a species is influenced by many environmental and biotic variables in its distributional range [10]. Each species has a unique combination of tolerances to various climatic factors, and any changes in these factors can cause potential changes in both the ecology and habitat of species and hence their geographical distribution [11,12]. Thus, we need to identify the potential effects of climate change in any area that is particularly vulnerable [13]. Rising temperatures are one of the most important climatic factors that can induce habitat shifts and/or population declines and are expected to become the primary threat to wildlife survival [14]. The average global temperature has increased by 0.74 °C in the last few decades and is expected to increase by another 1.4–5.8 °C in 2100 [15]. Consequently, almost 30% of plant and animal species are expected to be extinct by the end of this century [16].

Species distribution models (SDMs) are commonly used to determine conservation priorities by providing insights into the ecology and distributions of species [17] and by giving information for managing resources and habitats [11]. They have become crucial tools for determining how anthropogenic and natural environmental changes can affect species distributions. Biologists and managers can use SDMs to predict biological invasions and identify critical habitats, and they can decide where to translocate threatened species by anticipating their occurrence over time and space [18,19]. 

As with most agricultural insects (pests and pollinators), niche modelling studies of the family Bombyliidae, including *Spogostylum ocyale*, have received less attention than those devoted to insects of medicinal importance [20,21] or to other taxa, such as vertebrates [22]. To date and to our knowledge, only two studies [6,23] have been conducted on the distribution of bee flies in Egypt and the Middle East. The first analysed the distribution of all bee fly species based on their occurrence in the various ecological zones, while the second employed maximum entropy (Maxent) modelling to forecast the probable climatic niches of six threatened *Anthrax* species. The results showed that bee fly species richness was strongly correlated with plant diversity and that suitable habitats have vanished due to substantial changes in floral and faunal compositions over the last 50 years. 

The present study is based on *S. ocyale* records mainly from Egypt and eight neighbouring Middle Eastern countries (Palestine, Israel, Jordan, Iraq, Saudi Arabia, United Arab Emirates, Oman and Yemen). During the last 30 years, the third author has conducted a great number of collecting visits to various locations in Egypt and Saudi Arabia and has purposefully visited the same localities where bee flies, including *S. ocyale,* have been collected by prior authors. However, like many other bee flies, *S. ocyale* has become exceedingly rare or has disappeared from many of its historic habitats because of substantial changes in floral and faunal compositions in recent years, indicating that the species is endangered or vulnerable (at least in parts of its distribution range). Climate change, urbanisation and modern agricultural operations may be to blame for these changes, which have resulted in significant destruction of many animals’ native habitats.

Based on climatological and topographical data, the present study aims to develop environmental niche models and determine the areas with the best habitat appropriateness for the bee fly *S. ocyale*. In addition, we predict the effects of climate change on habitat suitability in 2071–2100 under two distinct climate change scenarios (SSP126 and SSP585) using five general circulation models (GCMs).

## 2. Materials and Methods

### 2.1. Research Area 

This study was carried out in Egypt and eight neighbouring Middle Eastern countries, including Palestine (West Bank), Israel, Jordan, Iraq, Saudi Arabia, United Arab Emirates, Oman and Yemen. The study covers approximately 4.5 million km^2^ of land.

### 2.2. Distribution Data for S. ocyale

A total of 88 *S. ocyale* records from nine countries were used in the study. These records were compiled using museum material and/or material collected by the third author through a series of collecting trips, primarily in Egypt and Saudi Arabia, between 1991 and 2020. Material from Efflatoun’s collection at Cairo University (EFC), the Entomological Society of Egypt’s collection (ESEC) and the third author’s personal collection were considered. For records from other countries, the following literature was used: Austen [24] for Palestine and Israel; Greathead [25] and Arabyat et al. [26] for Jordan; Zaitzev [27] for Iraq; Greathead [25] for the United Arab Emirates; Greathead [28] and Greathead [25] for Oman; and Greathead [25] for Yemen. In addition, Greathead [29,30] was the source for large numbers of records from around the Arabian Peninsula.

### 2.3. Environmental Data

Herein, 19 climatic variables and one topographical variable were downloaded from the WorldClim database (https://worldclim.org/, accessed on 20 July 2022) with a spatial resolution of 30 arc sec (~1 km) to assess current climatic conditions (Table 1). Environmental niche modelling for *S. ocyale* was performed according to the method described by Nasser et al. [6]. Briefly, the jackknifing function in Maxent was used to determine variables that showed any contribution to *S. ocyale* distribution. These variables were then subjected to the correlation test by SDMToolbox v2.5 (for ArcMap 10.7) to remove variables with high collinearity (Table S1) [6]. Consequentially, we used a set of seven most important bioclimatic layers that contribute to habitat suitability (Table 2). All these layers were clipped by ArcGIS software v10.7 to match the dimensions of the studied area and saved in ASCII grid format for use in Maxent. 

Five general circulation models (GFDL-ESM4, IPSL-CM6A-LR, MPI-ESM1-2-HR, MRI-ESM2-0 and UKESM1-0-LL) for the year 2085 (2071–2100) were downloaded from the CHELSA (Climatologies at high resolution for the earth’s land surface areas) database (https://chelsa-climate.org/, accessed on 25 July 2022) with a high resolution of 30 arc sec (~ 1 km) [31]. We used these models to predict the effects of climate change on *S. ocyale* according to the emissions scenarios ssp126; SSP1-RCP2.6 and ssp585; SSP5-RCP8.5. The five models used in this study are the primary ISIMIP3b models that are used compulsorily by the ISIMIP impact modelling team. These models are good representatives of the whole CMIP6 ensemble in terms of climate sensitivity and are structurally independent in terms of their ocean and atmosphere model components, and their process representation is good (GFDL-ESM4, MRI-ESM2-0 and UKESM1-0-LL) to fair (IPSL-CM6A-LR and MPIESM1-2-HR) [32]. Details of these climate models are listed in Table 3.

### 2.4. Habitat Suitability Model

To predict the current and future distributions of *S. ocyale*, the maximum entropy modelling was used with the Maxent software version 3.4.4 [33]. Maxent performs well with presence-only information and relatively few occurrence records [34,35], and it is broadly used in projecting range shifts of species distribution under climate change [36]. This technique generates mathematical models that describe the relationships between the probability of predicting a species and predictor variables and translates these models into potential distribution maps, indicating the environmental suitability of *S. ocyale*. The suitability values for a species habitat vary from 0 (unsuitable) to 1 (highly suitable) [37,38]. For the model, 25% of the occurrence records were assigned as the test data, and tenfold cross-validation (10 replicates) was used to enhance the model performance. To evaluate the accuracy of the generated model, we used two metrics: the area under the curve (AUC) of the receiver operating characteristics (ROC) curve [39] and the true skill statistics (TSS) [40]. The closer the test value is to 1, the better the discrimination is, and the more precise and informative the model [39,40].

## 3. Results

### 3.1. Evaluations of the Model and Its Importance to Variables 

**Figure 1:**
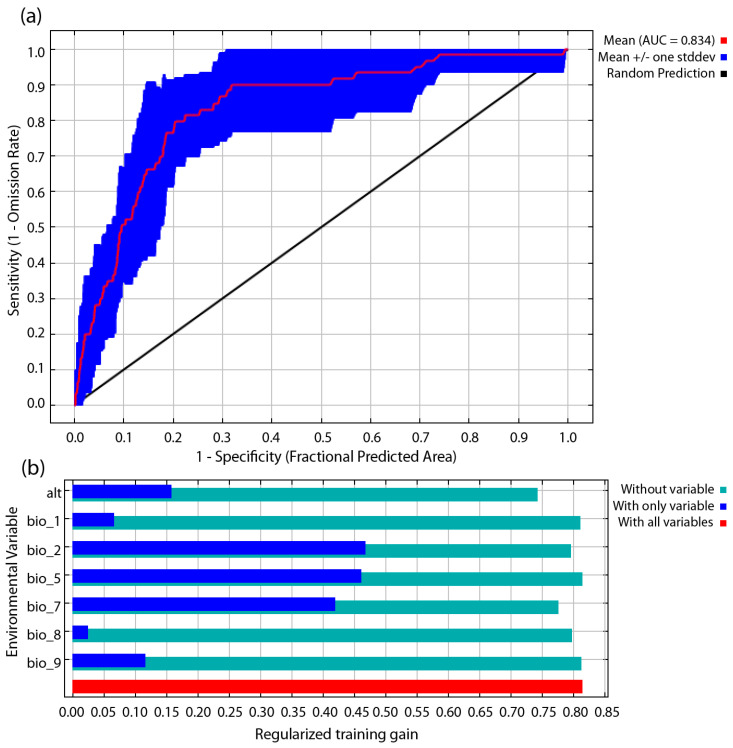
(**a**) Reliability test of the distribution model created for *Spogostylum ocyale* (values shown are averages over ten replicate runs). (**b**) Jackknife test results for Spogostylum ocyale, showing the relative predictive power of environmental variables. (Without variable)—the importance of the other environmental variables when this variable is omitted; (With only variable)—the importance of the environmental variable when used in isolation; (With all variables)—the importance of all environmental variables.

The performance for *S. ocyale* was satisfactory (AUC mean = 0.834; TSS mean = 0.606, Figure 1a and Table S2) and revealed a good potential distribution for *S. ocyale* featured by the selected factors. The jackknife test indicates that the distribution of *S. ocyale* was mainly affected by the maximum temperature of the warmest period (Bio5) and the temperature annual range (Bio7), and these variables contributed 34.4% and 32.5% to the species habitat model, respectively (Figure 1b and Table 2). The Maxent model was also collectively influenced by four other layers of climatic variables of 21.0% and one topographic variable (Alt) of 12.1% (Figure 1b and Table 2). Considering the permutation importance, the temperature annual range (Bio7) had the highest influence on the habitat model with 63.3% contribution, while altitude (Alt) and annual mean temperature (Bio1) contributed 15.9% and 9.9%, respectively (Table 2). The species response curve represents the relation between variable layers (climatic and topographic) and the suitability of the species habitat [38]. Based on the obtained response curves, *S. ocyale* prefers the maximum temperature of the warmest period from 20 to 35 °C, temperature annual range from 10 to 25 °C, annual mean temperature from 2 to 20 °C, mean diurnal range (max temp−min temp) from 3 to 12 °C and altitude from − 800 to 0 m and above 3000 m (very low and very high elevations) (Figure S1).

### 3.2. Current Potential Distribution of Spogostylum ocyale

**Figure 2:**
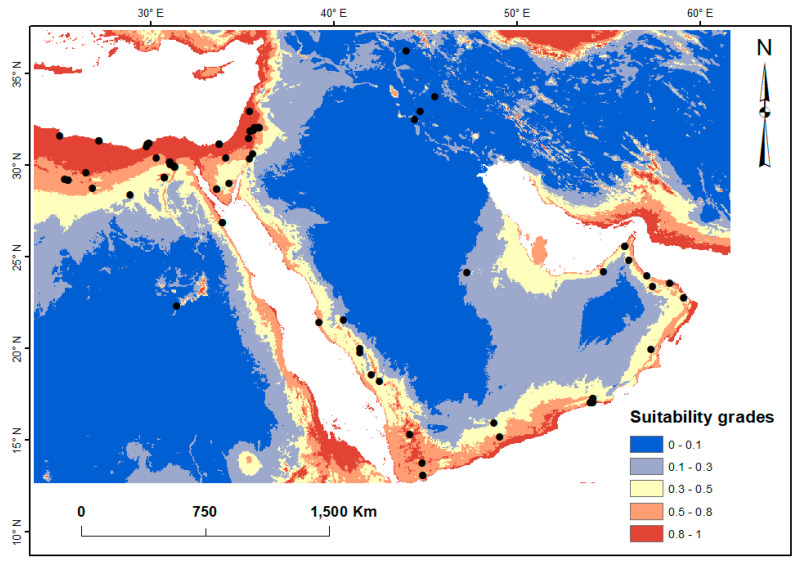
Occurrence records and potentially suitable climatic distribution of Spogostylum ocyale in Egypt and some Middle Eastern countries.

The distribution map of S. *ocyale* in Egypt and some Middle Eastern countries indicated 5.85%, 7.43% and 10.20% of the study localities, respectively, were identified as high, medium and general potential habitats (Figure 2). Generally, the predicted suitable habitats (high, medium and general) of S. *ocyale* were mainly situated at the coasts along the Mediterranean Sea, the coasts along the Red Sea, the Gulf of Suez coast, the Gulf of Aqaba coasts, the Gulf of Aden coasts, the Gulf of Oman coasts, and some areas in the coasts of Arabian Gulf. Apparently, the closer the locality is to the coastline, the higher the suitability is for this species. The average of the high-distribution areas was about 487 thousand km^2^ (5.85%), while the medium- and generally distributed areas were estimated at 618 (7.43%) and 848 (10.20%) thousand km^2^. In Egypt, the predicted distribution exhibited localities with high suitability for *S. ocyale* in the Coastal Strip, Nile Delta, North Sinai, and Red Sea coast (Figure 2).

### 3.3. Future Potential Distribution of Spogostylum ocyale

**Figure 3:**
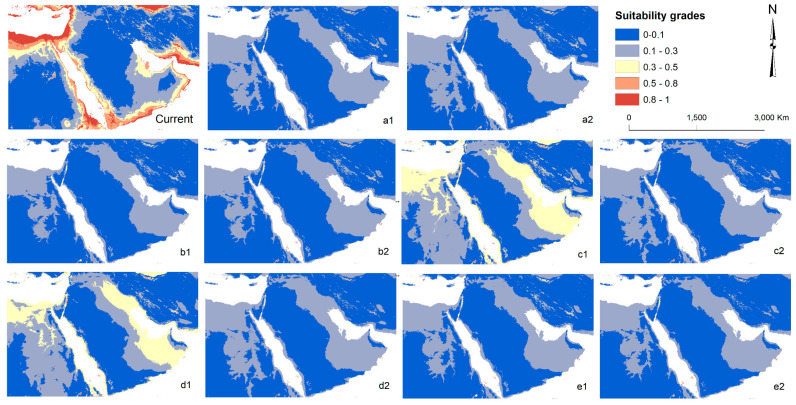
Predicted future habitat suitability of Spogostylum ocyale in Egypt and some Middle Eastern countries for the year 2085 (2071–2100) by five general circulation models (GCMs): a GFDL-ESM4, b IPSL-CM6A-LR, c MPI-ESM1-2-HR, d MRI-ESM2-0 and e UKESM1-0-LL (according to ssp126; SSP1-RCP2.6 emission scenario (1) and ssp585; SSP5-RCP8.5 emission scenario (2)).

The predicted distribution map of *S. ocyale* for the five primary models under SSP126 and SSP585 scenarios exhibits a progressive decline in the extent of suitable habitats with global climate warming (Figure 3). The total area of suitable distribution (high, medium and general suitability) for *S. ocyale* is expected to decline from 1.9 million km^2^ (23.5%) in 1970–2000 to 23 thousand km^2^ (0.27 ± 0.06%) on average in 2071–2100 for the five models under the two scenarios (SSP126 and SSP585) except for the MPI-ESM1-2-HR and MRI-ESM2-0 models under the SSP126 scenario. Thus, the unsuitable habitats for *S. ocyale* in Egypt and other countries are expected to increase by 23.2% in 2071–2100 at the expense of suitable habitat loss. For the MPI-ESM1-2-HR and MRI-ESM2-0 models, the potentially suitable total areas (19.72 and 18.6%, respectively) would not change much under the SSP126 scenario, yet the area of general potential habitat (0.3–0.5 suitability grade) alone represents about 98.8 and 98.9% of the suitable total areas for both models, respectively. These estimates predict that the distribution of suitable habitats for *S. ocyale* will diminish in the future, and species habitat loss will be relatively larger in SSP585 than in SSP126 (Figure 3), indicating an inverse relationship between the distribution of *S. ocyale* and the greenhouse gas emissions.

## 4. Discussion

Distribution modelling based on the environmental variables of known occurrence sites is a powerful tool in analytical biology, with applications in ecology, evolution, invasive species management, epidemiology and other fields [41]. Furthermore, this technique is important in biogeographic research, and various SDMs have been presented to support conservation decision making at the regional and global levels [19,42]. Here, the present and future distributions for the parasitoid bee fly *S. ocyale* were predicted using the maximum entropy model (Maxent). This algorithm has been broadly implemented in species distribution studies [42]. The model predicts the spatial distribution based on the suitable climatic factors of the species; it is believed that under known climatic conditions, the distribution of the species approaches reality as the entropy of the distribution increases [43]. This allowed us to determine which variables are informative in explaining the distribution of species and how they behaved [42]. Temperature is well known to be a climate factor that influences the abundance and distribution of insects [44,45]. Based on our models, the maximum temperature of the warmest period (Bio5) and temperature annual range (Bio7) are predicted as the most important variables that affect the distribution of *S. ocyale*. These results are consistent with previous literature on different insects, e.g., [6,46,47,48]. Other variables are less important in shaping the distribution of *S. ocyale*. However, altitude is a significant factor impacting the distribution of species as well [6,49]. In our research, the response curves revealed an inverse correlation between temperature and species occurrence (Figure S1). In addition, it demonstrated that the parasitoid bee fly can be found in profiles of minimum temperatures that include both extremely low- and high-altitude sites (10–25 °C). Our finding of maximum temperature of the warmest period (Bio5) and temperature annual range (Bio7) as having the topmost relative contribution in the model implies that the distribution of *S. ocyale* is influenced mainly by temperature, especially considering that the climatic zones of the Middle Eastern countries range from arid and semiarid to temperate climatic zones, where precipitation is scarce throughout the year [50]. In comparison, variables related to precipitation or its variations were found to have a substantial influence on the predicted distribution of parasitoids in tropical regions, such as *Comperiella calauanica* in the Philippines [11].

Recent developments in ecological modelling have improved our capacity to predict and estimate the suitable localities of species with environmental variables and can fill in information gaps when determining the need for conservation [51]. In the present study, the Maxent model predicted suitable zones for the parasitoid bee fly in the Middle East region. According to the habitat distribution map (Figure 2), coastal regions, with a warm summer and cold winter, were high to medium suitable areas for the parasitoid. As a result, arid to semiarid regions (with temperatures above 35 °C) with very low humidity may not be the preferred habitats for *S. ocyale* (Figure S1). The potential distribution for this parasitoid in Egypt closely matches what has been documented by Nasser et al. [6] in an environmental niche modelling study on six bee fly species that are thought to be threatened. The results of this research showed different habitat suitability and different predictor (altitude, temperature and precipitation) contributions for each species. However, the predicted distributions of two (*Anthrax chionanthrax* and *A. melanista*) of these bee flies were largely dominated by temperature and showed areas with high suitability in the Sinai, Red Sea coast, Coastal Strip, some areas in the Western Desert and Eastern Desert, and Lower Nile Valley and Delta.

Several recent studies observed the ability to predict the distribution of the host species by the distribution model of its parasitic wasp, when the suitable localities for the parasitoid were consistent with localities of its hosts, e.g., [11,52,53]. Here, the parasitoid bee fly has several host species (immature stages of solitary bees and wasps, egg pods of Acrididae and pyralid cocoons) and is not highly host specific. Although climate is the primary factor influencing its distribution, more studies are required on this species to better understand its behaviour and relationship with its hosts in the field. 

Biodiversity patterns and changes in the Earth’s climate are intimately intertwined [54]. In response to long-term climate change, the geographic distributions of species are shrinking, expanding, shifting or fragmenting [51]. Moreover, it is anticipated to result in species experiencing phenological inconsistency and facing new competition and community structures [54]. With ectotherms in particular, insects are most vulnerable as their biochemical and physiological processes are firmly controlled by ambient temperature [55,56]. Our analyses exhibit strong indications for a possible extensive reduction in distribution patterns for the parasitoid bee fly. Except for the MPI-ESM1-2-HR and MRI-ESM2-0 models under the SSP126 scenario (Figure 3: c1, d1), the parasitoid was predicted to lose about 98.7% of its range under both SSP scenarios, putting it on the brink of extinction. The future distribution maps almost show an agreement in predicted suitability of habitats among the five general circulation models under low (SSP126) and high (SSP585) emission scenarios (Figure 3). A closely related bee fly species (*A. trifasciatus*) exhibited a similar pattern of habitat degradation in the St. Catherine area for the year 2070 under two different future emission scenarios [6]. Given that the geographical distribution of *A. trifasciatus* in Egypt is limited only to the Sinai Peninsula, it can be classified as a potentially endangered species.

Despite the limitations of SDMs, the threats posed by climate change to species will certainly be compounded by the continued destruction of its remaining habitats. In Egypt, during the last 50 years, dramatic changes occurred in the floral and faunal assemblages in the parasitoid’s highly suitable areas (e.g., Delta and Coastal Strip). These changes may be accounted for by urbanisation together with other anthropogenic activities that cause severe reduction and degradation of the parasitoid’s habitats. However, there are some areas where urban sprawl has not yet reached, and they still maintain their natural fauna and flora. Sinai is one of these localities that can contribute significantly to the conservation of such rare species, especially because it has one of the most important natural reserves (St. Catherine protectorate) in Egypt. Therefore, implementing the appropriate conservation actions will be applicable.

The outcomes of climate change and species adaptation over hundreds of thousands of years are not comparable to the changes taking place within a century in terms of biodiversity [54]. The adaptation of the *S. ocyale* species may not be fast enough to keep pace with the rate of habitat loss and degradation predicted during this study by the year 2085. Furthermore, the implications might be worse than what we estimated here if humans are unable to reduce carbon emissions. 

In addition to climate, which is thought to be the main predictor of species distribution at a wide landscape scale [57], biotic variables, such as biotic interactions, vegetation types and land use, should be taken into consideration to enhance model performance and to urge an improved understanding of the effects of global warming on species distributions [48,57]. Accordingly, the potential distribution of the parasitoid S. ocyale in the studied region represents its fundamental niche of actual environmental tolerances and not its realized niche as a result of biotic variables. Thus, conservation actions based solely on this distribution would be less restrictive and falsely optimistic.

## 5. Conclusions

In this study, we developed an SDM for the parasitoid bee fly *S. ocyale* in the Middle East based on climatic and topographical variables of known occurrence sites. Moreover, the results exhibit shrinkage in the future suitable habitats predicted for the parasitoid. Finally, we trust this is a potent evidence-based assumption that should be used to support conservation planning.

## Figures and Tables

**Table 1:** Environmental and topographical variables used in the distribution modelling process of *Spogostylum ocyale*.

Variable Code	Description	Unit
Bio1	Annual mean temperature	°C
Bio2	Mean diurnal range (max temp−min temp) (monthly average)	°C
Bio3	Isothermality (Bio1/Bio7) × 100	°C
Bio4	Temperature seasonality (coefficient of variation)	°C
Bio5	Max temperature of warmest period	°C
Bio6	Min temperature of the coldest period	°C
Bio7	Temperature annual range	°C
Bio8	Mean temperature of wettest quarter	°C
Bio9	Mean temperature of driest quarter	°C
Bio10	Mean temperature of warmest quarter	°C
Bio11	Mean temperature of coldest quarter	°C
Bio12	Annual precipitation	mm
Bio13	Precipitation of wettest period	mm
Bio14	Precipitation of driest period	mm
Bio15	Precipitation seasonality (coefficient of variation)	mm
Bio16	Precipitation of wettest quarter	mm
Bio17	Precipitation of driest quarter	mm
Bio18	Precipitation of warmest quarter	mm
Bio19	Precipitation of coldest quarter	mm
Alt	Altitude in degrees	m

**Table 2:** Percent contributions of the bioclimatic and topographic variables in the Maxent models for *Spogostylum ocyale* (10 replicated runs).

Variables	% Contribution	Permutation Importance
Bio5	34.4	0
Bio7	32.5	63.3
Bio2	14.3	4.1
Alt	12.1	15.9
Bio8	6.1	6.8
Bio1	0.5	9.9
Bio9	0.1	0

**Table 3:** Details of the primary five general circulation climate models used for the prediction of future habitat suitability of *Spogostylum ocyale*.

Model ID	Institution	Native Resolution	Ensemble Member	Priority ^a^
GFDL-ESM4	National Oceanic and Atmospheric Administration, Geophysical Fluid Dynamics Laboratory, USA	288 × 180	r1i1p1f1	1
IPSL-CM6A-LR	Institute Pierre Simon Laplace, France	144 × 143	r1i1p1f1	4
MPI-ESM1-2-HR	Max Planck Institute for Meteorology, Germany	384 × 192	r1i1p1f1	3
MRI-ESM2-0	Meteorological Research Institute, Japan	320 × 160	r1i1p1f1	5
UKESM1-0-LL	Met Office Hadley Centre, UK	192 × 144	r1i1p1f2	2

^a^ The priority of models follows the suggestions of the ISIMIP3b protocol.

## Data Availability

All data generated or analysed during this study are included in this article.

## Supplementary Materials

The following supporting information can be downloaded at: https://www.mdpi.com/article/10.3390/insects14020120/s1, Table S1: Correlation test of variables that showed any contribution to *Spogostylum ocyale* distribution by SDMToolbox v2.5 in ArcMap 10.7 (Universal tool; Remove highly correlated variables). Table S2: Performance evaluation of the MaxEnt model using area under the ROC curve “AUC” and true skilled statistics (TSS). Figure S1: *Spogostylum ocyale* response curves in relation to bioclimatic predictors: values shown are average over ten replicate runs.
