# Supplementary material for: Effects of Climatic Change on Potential Distribution of Spogostylum ocyale (Diptera: Bombyliidae) in the Middle East Using Maxent Modelling
Source: Insects. 2023 Jan 24;14(2):120. doi: 10.3390/insects14020120 (PMC9960050; doi:10.3390/insects14020120)
Supplement: Supplementary file 1 [file insects-14-00120-s001.zip › insects-2129054-supplementary/Figure S1.pdf]

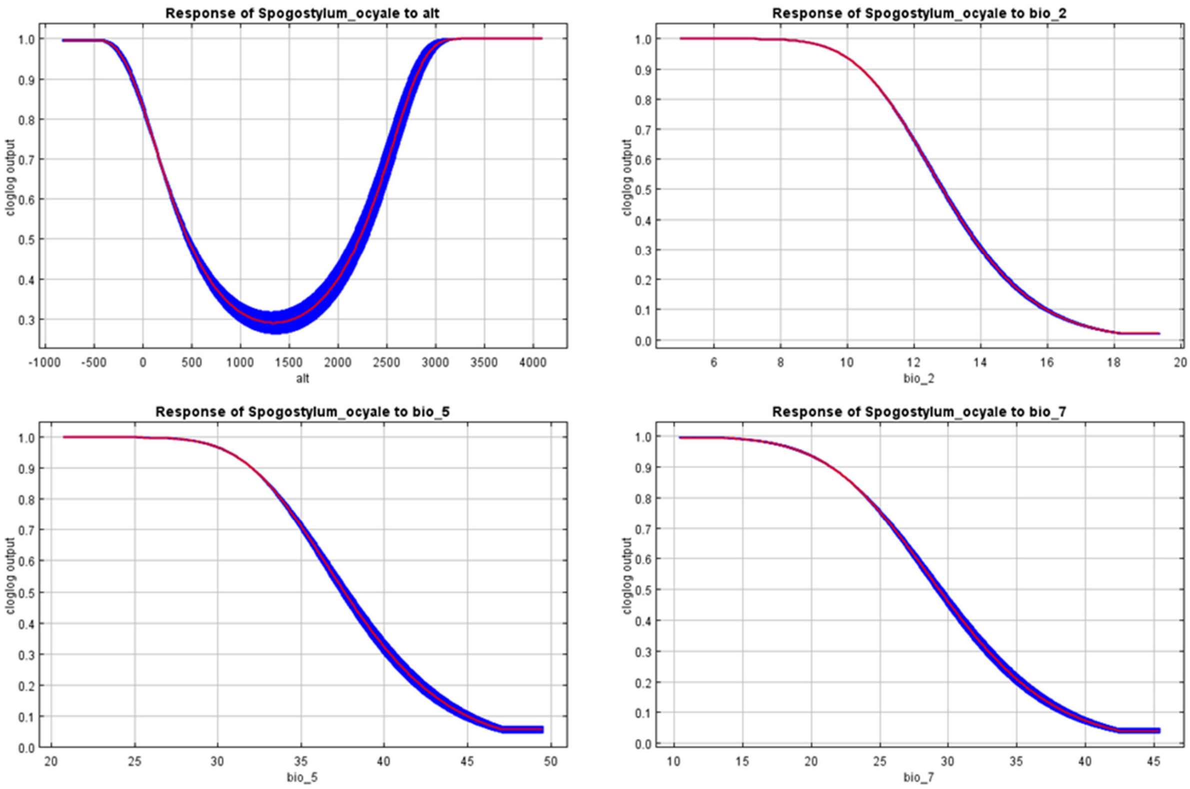

Figure S1. *Spogostylum ocyale* response curves in relation to bioclimatic predictors: values shown are average over ten replicate runs.
