# Supplementary material for: Effects of Climatic Change on Potential Distribution of Spogostylum ocyale (Diptera: Bombyliidae) in the Middle East Using Maxent Modelling
Source: Insects. 2023 Jan 24;14(2):120. doi: 10.3390/insects14020120 (PMC9960050; doi:10.3390/insects14020120)
Supplement: Supplementary file 1 [file insects-14-00120-s001.zip › insects-2129054-supplementary/Table S1.pdf]

Table S1. Correlation test of variables that showed any contribution to *Spogostylum ocyale* distribution by SDMToolbox v2.5 in ArcMap 10.7 (Universal tool; Remove highly correlated variables).

variables	Bio2	Bio3	Bio4	Bio5	Bio6	Bio7	Bio8	Bio9	Bio10	Alt
Bio1	0.126	0.544	-0.55	0.725	0.913*	-0.469	0.851*	0.293	0.838*	-0.628
Bio2		0.322	0.098	0.414	-0.149	0.449	0.176	-0.118	0.183	-0.0009
Bio3			-0.890*	0.044	0.664	-0.680	0.707	-0.351	0.067	-0.158
Bio4				0.122	-0.784	0.925*	-0.712	0.338	-0.013	0.188
Bio5					0.424	0.246	0.437	0.589	0.950*	-0.541
Bio6						-0.773	0.835*	0.149	0.595	-0.553
Bio7							-0.587	0.252	0.029	0.212
Bio8								-0.106	0.538	-0.393
Bio9									0.603	-0.334
Bio10										-0.624

Shaded variables were removed because of high cross-correlations. The priority in our choice between correlated variables was for those represent the original input climate data and are not derived from several layers or a subset of the data (such as: Bio1 and Bio2). * The threshold for correlation was 0.8.
