# Supplementary material for: Effects of Climatic Change on Potential Distribution of Spogostylum ocyale (Diptera: Bombyliidae) in the Middle East Using Maxent Modelling
Source: Insects. 2023 Jan 24;14(2):120. doi: 10.3390/insects14020120 (PMC9960050; doi:10.3390/insects14020120)
Supplement: Supplementary file 1 [file insects-14-00120-s001.zip › insects-2129054-supplementary/Table S2.pdf]

Table S2. Performance evaluation of the MaxEnt model using area under the ROC curve “AUC” and true skilled statistics (TSS).

Model replicate	AUC[1]		True Skill Statistic (TSS)[2]
	Training	Test	
1	0.846	0.8416	0.686
2	0.835	0.93	0.862
3	0.853	0.7781	0.467
4	0.841	0.8389	0.566
5	0.868	0.6268	0.485
6	0.853	0.785	0.578
7	0.842	0.8946	0.694
8	0.84	0.9141	0.657
9	0.844	0.8727	0.555
10	0.847	0.8569	0.509
Average ± SD	0.8471 ± 0.009	0.834 ± 0.088	0.606 ± 0.120

[1]The area under the curve (AUC) has scores varying between 0 and 1, and values ≥0.7 are considered acceptable [39].

[2]True skilled statistics (TSS) scores vary from -1 to 1, and values ≥0.5 indicate acceptable models [40].
